# Beyond Respiration: Rethinking CO_2_ as a Regulator of Cellular Signaling in Microenvironmental Stress and Disease

**DOI:** 10.3390/ijms26125796

**Published:** 2025-06-17

**Authors:** Masahiko Shigemura

**Affiliations:** Division of Thoracic Surgery, Feinberg School of Medicine, Northwestern University, Chicago, IL 60611, USA; masahiko.shigemura@northwestern.edu; Tel.: +1-312-503-5033

## 1. Editorial: New Advances in Hypercapnia

Carbon dioxide (CO_2_) is a chemically simple molecule with essential roles in biology. Like oxygen, it is fundamental to life. A healthy adult produces approximately 300 L of CO_2_ per day, which is efficiently exhaled through normal ventilation [1]. Remarkably, the average human body stores about 130 L of CO_2_, highlighting its central role in maintaining acid–base balance and cellular function. Cells continuously monitor fluctuations in CO_2_ levels through evolutionarily conserved pathways that elicit adaptive responses to preserve homeostasis [2].

Hypercapnia, an elevation in CO_2_ in blood and tissues, commonly arises in advanced lung diseases. Emerging evidence suggests that various cell types sense hypercapnic stress through mechanisms that remain incompletely understood. Reported sensing pathways activate distinct signaling cascades, some of which disrupt cellular and tissue homeostasis and may contribute to disease progression [2].

Beyond its physiological significance, CO_2_ has also become a global environmental concern—both on Earth and in space. Natural geological processes such as volcanism contribute to atmospheric CO_2_ levels [3]. Since the Industrial Revolution, human activities have driven a substantial and sustained increase in atmospheric CO_2_ concentrations, making it a major contributor to climate change. Concurrently, the rapid advancement of space exploration has brought new attention to the physiological impacts of spaceflight on human health. Notably, although the oxygen and nitrogen concentrations in astronauts’ breathing zones aboard the International Space Station (ISS) remain within normal terrestrial ranges, CO_2_ levels are typically at least ten times higher than those on Earth [4]. Looking ahead, exposure to such “environmental” hypercapnia may represent an underappreciated risk to human health under specific conditions.

Given its broad biological and environmental impacts, a comprehensive understanding of CO_2_ biology and the pathological consequences of hypercapnia is more important than ever. In this Research Topic, an international consortium of investigators present original studies that provide novel insights into CO_2_ sensing and signaling, as well as the acute and chronic effects of hypercapnia on diverse cell types, including immune cells, epithelial cells, and cancer cell lineages. The author provides an overview of the key findings and thematic advances presented in these contributions and outlines potential directions for future research.

## 2. Original Research Articles

Each of the following studies examines distinct aspects of hypercapnia biology—from molecular sensing/signaling mechanisms to clinical implications—beginning with a focus on post-translational modifications.

Gannon HG et al. [5] significantly advance our understanding of how cells detect and respond to elevated CO_2_ levels through post-translational modifications (PTMs). Using innovative chemical approaches, the authors identified a carbamate PTM on lysine 9 (K9) of *Arabidopsis* nucleoside diphosphate kinase 1 (AtNDK1), a highly conserved enzyme involved in nucleotide metabolism. Notably, they show that not all carbamate PTMs are functionally relevant, challenging the assumption that CO_2_ binding uniformly contributes to signaling. By distinguishing between functional and non-functional carbamate sites, the study refines our understanding of CO_2_ sensing as a selective, rather than universal, process. These findings open new avenues for exploring how organisms discriminate between incidental CO_2_ interactions and those of adaptive significance. This work represents both conceptual and technical leaps in decoding the biochemical logic of CO_2_ responsiveness.

Phelan DE et al. [6] show that orphan nuclear receptors NR4A2 and NR4A3 play a role as key transcriptional regulators in the cellular response to acute hypercapnic stress in monocytes. These nuclear receptors are early-response genes activated by various stimuli, and they modulate monocyte differentiation. The authors demonstrate that NR4A2 and NR4A3 mediate distinct and overlapping gene expression responses to “buffered” hypercapnia (i.e., with physiological pH), including pathways related to mitochondrial function and heat shock protein regulation. Using targeted knockdown approaches and transcriptomic analyses, the study highlights NR4A2 and NR4A3 as context-specific modulators of CO_2_ sensitivity. Notably, the work reveals a core set of CO_2_-responsive genes independent of NR4A2/3, suggesting the involvement of additional conserved transcriptional regulators. Through this mechanistic dissection, the study advances our understanding of how hypercapnia shapes monocyte function and implicates the NR4A family in the broader transcriptional landscape of CO_2_ responses.

Zohar N et al. [7] shed light on the underexplored role of hypercapnia in the tumor microenvironment of pancreatic cancer. The authors demonstrate that chronic hypercapnia promotes an aggressive phenotype in pancreatic ductal adenocarcinoma (PDAC) cells, characterized by enhanced proliferation, migration, invasion, and chemotherapy resistance. Through transcriptomic analyses, the study reveals that hypercapnia activates key oncogenic pathways, including epithelial-to-mesenchymal (EMT) transition, extracellular matrix remodeling, and hypoxia-associated growth factor signaling. Notably, the study identifies SIAH3, an understudied E3 ligase family member, as a hypercapnia-responsive gene with potential prognostic significance in PDAC. These findings suggest hypercapnia as a distinct microenvironmental regulator of cancer behavior and highlight SIAH3 as a candidate target for further investigation. This work pioneers the conceptual framework for considering hypercapnia as a novel modulator of tumor progression and a potential therapeutic axis.

Chen F et al. [8] uncover a novel mechanism by which hypercapnia enhances influenza A virus (IAV) infection in airway epithelial cells. The authors demonstrate for the first time that exposure to high CO_2_ drives IAV adhesion, internalization, and replication by increasing cellular cholesterol through the activation of the mTOR/Akt/SREBP2 signaling axis. Notably, the pharmacologic blockade of cholesterol synthesis or mTOR/Akt signaling specifically counteracts hypercapnia-induced viral propagation, offering a new therapeutic angle. This work highlights hypercapnia as a disease-relevant modifier of host susceptibility to viral infection beyond its traditionally understood physiological roles. By linking CO_2_ signaling to lipid metabolism and viral pathogenesis, the study provides a mechanistic explanation for worse infectious outcomes in hypercapnic patients. These findings open the door to targeted therapies that could mitigate infection risk in patients with chronic hypercapnia.

## 3. Conclusions and Future Directions

This Research Topic brings together a diverse body of work that advances our understanding of elevated CO_2_ sensing and its physiological and pathological effects. Collectively, the contributions highlight that hypercapnia is sensed through both acute and chronic mechanisms involving complex molecular pathways, including post-translational modifications, transcriptional regulation, and diverse signaling cascades. The consequences of elevated CO_2_-driven signaling appear to be highly context-dependent, with both adaptive and maladaptive outcomes depending on the cell type, duration of exposure, and underlying disease state.

The studies featured here strongly advocate for continued research into the molecular and cellular mechanisms of elevated CO_2_ sensing, with implications for human health, disease management, and planetary science. As atmospheric and enclosed-environment CO_2_ levels continue to rise, future studies integrating systems biology, clinical data, and environmental modeling will be critical in fully deciphering the complex role of hypercapnia in human health and disease.

## References

[1] Cherniack N.S., Longobardo G.S. (1970). Oxygen and carbon dioxide gas stores of the body. Physiol. Rev..

[2] Cummins E.P., Strowitzki M.J., Taylor C.T. (2020). Mechanisms and Consequences of Oxygen and Carbon Dioxide Sensing in Mammals. Physiol. Rev..

[3] Boada L.D., Simbana-Rivera K., Rodriguez-Perez C., Fuentes-Ferrer M., Henriquez-Hernandez L.A., Lopez-Villarrubia E., Alvarez-Leon E.E. (2024). Assessing the hidden dangers of volcanic CO_2_ exposure: A critical review of health impacts. Front. Public Health.

[4] Sampige R., Ong J., Waisberg E., Berdahl J., Lee A.G. (2025). The hypercapnic environment on the International Space Station (ISS): A potential contributing factor to ocular surface symptoms in astronauts. Life Sci. Space Res..

[5] Gannon H.G., Riaz-Bradley A., Cann M.J. (2024). A Non-Functional Carbon Dioxide-Mediated Post-Translational Modification on Nucleoside Diphosphate Kinase of *Arabidopsis thaliana*. Int. J. Mol. Sci..

[6] Phelan D.E., Reddan B., Shigemura M., Sznajder J.I., Crean D., Cummins E.P. (2024). Orphan Nuclear Receptor Family 4A (NR4A) Members NR4A2 and NR4A3 Selectively Modulate Elements of the Monocyte Response to Buffered Hypercapnia. Int. J. Mol. Sci..

[7] Zohar N., Maguire R., Khalilieh S., Jain A., Bosykh D., Bowne W.B., Lavu H., Yeo C.J., Nevler A. (2025). Gene Expression Profiling of Pancreatic Ductal Adenocarcinoma Cells in Hypercapnia Identifies SIAH3 as a Novel Prognostic Biomarker. Int. J. Mol. Sci..

[8] Chen F., Matsuda A., Sporn P.H.S., Casalino-Matsuda S.M. (2025). Hypercapnia Increases Influenza A Virus Infection of Bronchial Epithelial Cells by Augmenting Cellular Cholesterol via mTOR and Akt. Int. J. Mol. Sci..

